# Case Report and Review of Literature: Thyroid Metastases From Breast Carcinoma

**DOI:** 10.3389/fendo.2021.631894

**Published:** 2021-03-12

**Authors:** Yichao Wang, Shengliang Zhou, Boyang Yu, Ping Zhou, Jingqiang Zhu, Tao Wei, Zhihui Li

**Affiliations:** ^1^ Department of Thyroid & Parathyroid Surgery Center, West China Hospital, Sichuan University, Chengdu, China; ^2^ West China School of Medicine, West China Hospital, Sichuan University, Chengdu, China; ^3^ Department of Sonography, West China Hospital, Sichuan University, Chengdu, China; ^4^ Department of Pathology, West China Hospital, Sichuan University, Chengdu, China

**Keywords:** thyroid metastases, breast carcinoma, diagnosis, therapy, immunohistochemistry

## Abstract

**Rationale:**

The thyroid is a rare site for distant metastases from breast carcinoma. The incidence of thyroid metastases in fine needle aspiration biopsy (FNAB) was less than 0.2%.

**Patient concerns:**

We report a case of 54-year-old woman with a history of breast carcinoma presented with diffuse scattered microcalcifications in thyroid and enlarged bilateral cervical lymph nodes detected on ultrasound (US). Physical examination of the patient revealed firm and enlarged thyroid lobes.

**Diagnoses:**

FNAB and immunohistochemistry (IHC) of the thyroid lesion confirmed the thyroid metastases from breast cancer.

**Interventions and Outcomes:**

Due to the comorbidities of breast carcinoma metastases to the right axillary, cervical lymph nodes and left chest wall, the patient received chemotherapy. After a follow-up of 19 months, the patient was alive without any new distant metastases.

**Lessons:**

Our case highlights that thyroid metastases should be considered in a patient combined with thyroid lesions and a history of breast carcinoma. IHC played an important role in differentiating thyroid metastases from primary thyroid cancer.

## Introduction

Thyroid metastases from breast cancer are unusual, accounting for less than 0.2% of thyroid fine needle aspiration biopsy (FNAB) (1, 2). It is well known that the most frequent distant metastases of breast cancer are bone and visceral organs (3). Generally, the common origins of thyroid metastases seem to be breast cancer and lung cancer in autopsy cases (4). However, in clinical cases, the most common primary site is the kidney, followed by the breast (5). Recently, with the advent of advanced diagnostic methods such as FNAB and immunohistochemistry (IHC), thyroid metastases have been reported increasingly. Although FNAB can help evaluate benign and malignant thyroid lesions, it may be inefficient in differentiating metastatic thyroid lesion by FNAB.

Here, we presented a case of thyroid metastases from breast carcinoma diagnosed by FNAB and immunochemistry.

## Case Reports

A 54 year old woman presented to the West China Hospital, Sichuan University for evaluation of the thyroid nodule and enlarged bilateral cervical lymph nodes detected on ultrasound (US). She denied any symptoms related to the thyroid disease. She had a personal history of invasive ductal carcinoma in the left breast (ER negative, PR negative, GATA3 positive and HER2 positive). She underwent left mastectomy and axillary lymphadenectomy, followed by chemotherapy with epirubicin, cyclophosphamide, dexrazoxane, and trastuzumab. A core needle biopsy of right axillary adenopathy and mass in the left chest wall revealed metastatic breast carcinoma 2 years after surgery.

On physical examination of the thyroid, firm and enlarged thyroid lobes were identified. A 1 cm mass in the upper outer quadrant of the right breast and a 5 cm mass in the left chest wall were found. A 2 cm right axillary adenopathy were found. There were no palpable lymph nodes in the cervical region.

**Figure 1:**
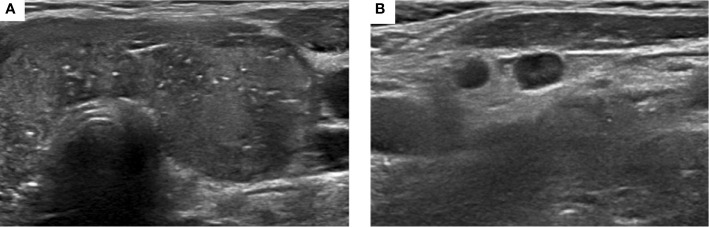
Ultrasound of thyroid showed heterogeneous enlargement and diffuse scattered microcalcifications **(A)**, and enlarged cervical lymph nodes **(B)**.

US showed heterogeneous enlargement and diffuse scattered microcalcifications in both lobes of the thyroid (**Figure 1**). A 0.7 cm hypoechogenic, well circumscribed nodule was also found in the left lobe of the thyroid. Right axillary and bilateral cervical lymph nodes were detected on US. The US results showed multiple lymph nodes in the lateral cervical region, the right was about 1.1 cm and the left was about 1 cm. Some of the dermal medulla was poorly demarcated, and dotted blood signals were seen inside the lymph modes. Thyroid function was normal.

**Figure 2:**
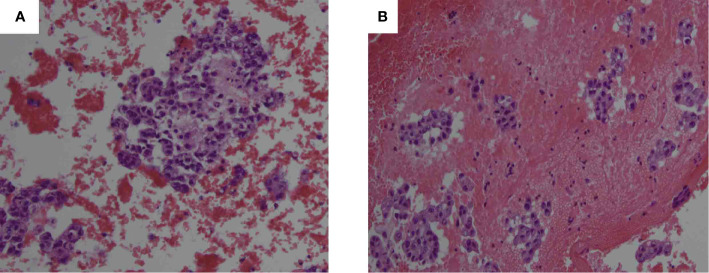
Hematoxylin and eosin (HE) stained FNAB of the right thyroid lobe (**A**, 200× magnification) and left thyroid lobe (**B**, 200× magnification) revealed malignant epithelial cells.

**Figure 3:**
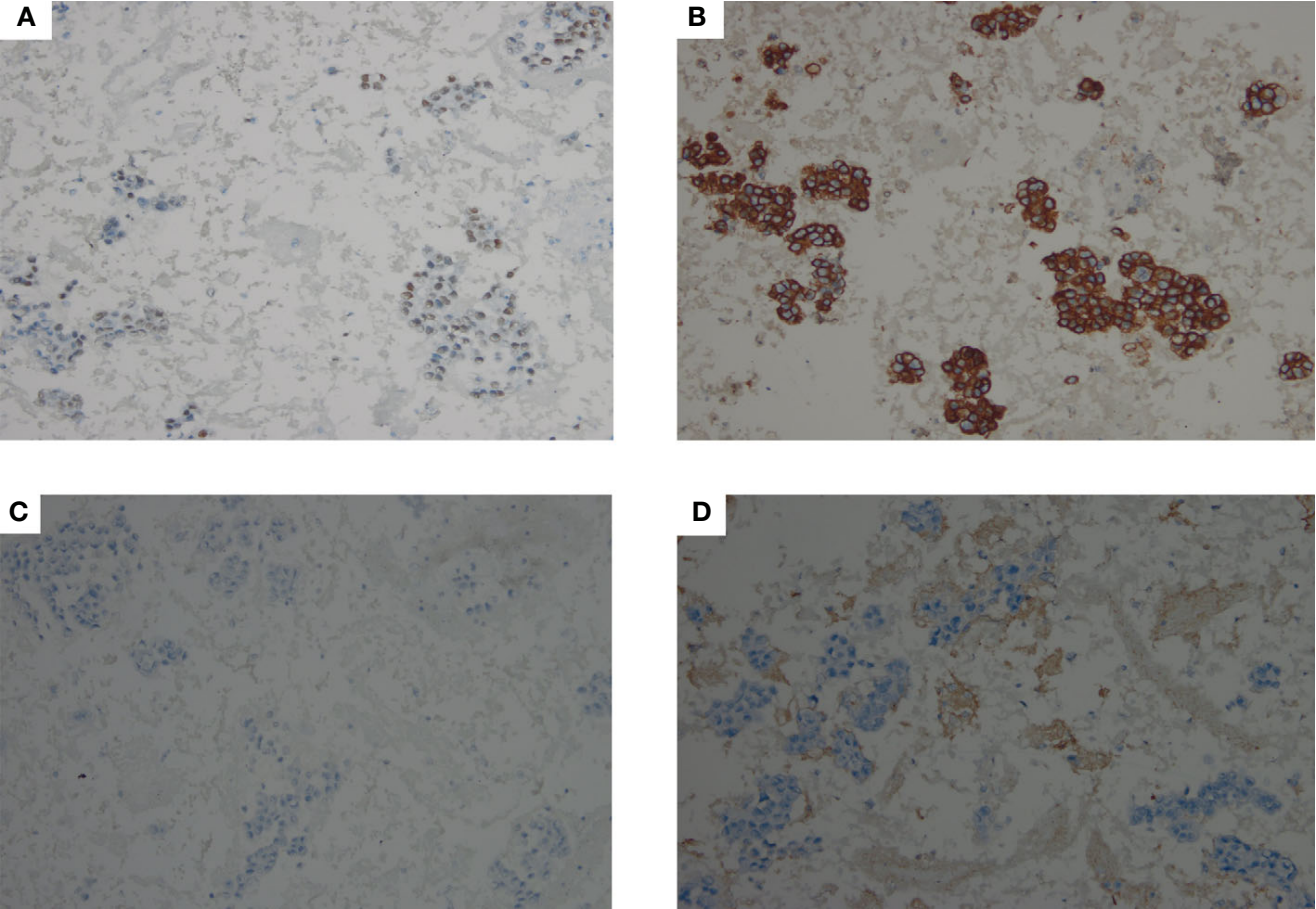
IHC staining showed that malignant cells in thyroid were positive for GATA3 (**A**, 200× magnification) and HER2 (**B**, 200× magnification), and negative for TTF1 (**C**, 200× magnification), TG (**D**, 200× magnification).

FNAB of bilateral lobes of the thyroid (**Figure 2**) and cervical lymph nodes demonstrated malignant epithelial cells. Immunohistochemical staining of thyroid (**Figure 3**), which exhibited positive GATA3 and HER2, and negative TTF1, TG, GCDFP15, PR and ER, confirmed breast carcinoma metastases to the thyroid.

Computed tomography (CT) showed no evidence of metastatic lesion in the brain, lung, liver and bone. Given the comorbidities of right axillary adenopathy and left chest wall metastases, the patient received chemotherapy. Because of financial difficulties, paclitaxel liposome and trastuzumab were administered in the patient. At the follow-up, the patient was alive at 19 months post-thyroid metastases diagnosis. Assessment by CT did not reveal any new distant metastases.

This study was approved by the ethics committee of the West China Hospital, Sichuan University, and written informed consent was provided by the patient for publication of this case publication.

## Discussion

To the best of our knowledge, the thyroid is an uncommon site of metastatic cancer. This may be attributed to the fast blood flow of thyroid, abundant oxygen and iodine (6). The incidence of thyroid metastases ranges from 1.25% to 24% in autopsy studies (7). But the incidence of thyroid metastases was lower in clinical series than autopsy studies (1, 8). It has been demonstrated that breast was the secondly frequent primary cancer site for thyroid metastases (3, 8). Currently, the etiology of thyroid metastases from primary carcinoma is not clarified. One study speculated that decreased oxygen and iodine resulted by local thyroid diseases (i.e., thyroiditis and goiter) may contribute to the genesis of thyroid metastases (9).

The clinical presentation of thyroid metastases from breast cancer were similar to primary thyroid cancer, such as no symptoms, palpable neck mass, thyroid lesions detected on imaging examination and compression symptoms. Some studies reported that patients with thyroid metastases from breast cancer were symptomless and presented malignant thyroid nodules by ultrasound (1, 3). Similar clinical manifestations were reported by Owens et al. (10) and Pensabene et al. (4). In our case, the patient also presented no symptoms related to the thyroid disease. Other studies showed patients with thyroid metastases from breast cancer demonstrated dysphagia and dyspnea (5–7, 11). In addition, the US features of thyroid metastases from breast cancer are not specific. Zhou et al. (1) reported that six patients showed heterogeneous echogenicity with scattered microcalcification, and two patients had hypoechoic solid nodule on US. Pensabene et al. (4) reported that one case showed enlarged thyroid with thyroid nodules on US. Similar thyroid nodule on US was reported by Magers et al. (2), Lacka et al. (6) and Owens et al. (10). Above US features also seen in our case and have been described previously. Therefore, there are no specific properties that clinically distinguish thyroid metastases from primary thyroid cancer.

Cytologic examination using FNAB is highly sensitive and specific in demonstrating a malignant thyroid nodule. But it is sometimes difficult to show the origin of the metastatic cancer (4). Owens et al. (10) reported that FNAB of thyroid metastases from breast cancer demonstrated malignant epithelial cells with enlarged nuclei and irregular nuclear contours, but in the absence of intranuclear grooves and pseudoinclusions. Magers et al. (2) found that metastatic thyroid cancer from breast cancer can cytologically and morphologically mimic primary thyroid cancer on FNAB. Additionally, metastatic breast cancer cells in the thyroid, which mimicked C cell hyperplasia and medullary thyroid carcinoma, were observed in the case of Ghias et al. (5). In our case, FNAB of thyroid showed malignant cells with enlarged nuclei.

It has been documented that immunohistochemical stains can play a critical role in differentiating between thyroid metastases and primary thyroid cancer (12). As noted, primary differentiated thyroid cancer commonly is TG, TTF-1 and PAX8 positive (12). Calcitonin, involved in the parafollicular cells, is associated with medullary thyroid carcinoma (13). In contrast, above immunohistochemical markers were negative for thyroid metastases (5, 14). On the other hand, considering the history of breast cancer, the immunoprofile of breast cancer such as ER, PR, HER2, GATA3, and GCDFP15, should be used to confirm the origin from breast cancer metastasis. Previous studies have demonstrated that GATA3 and GCDFP15 expression were positively associated with breast carcinoma and breast cancer metastasis (15, 16). GATA3 is superior to GCDFP15 in determining the breast origin (17, 18). Ghias et al. (5) reported that thyroid metastases cells were positive for ER, GATA3, and were negative for GCDFP, TTF-1, TG, and calcitonin. Similar result is also observed in our case. Bourcier et al. (19) showed thyroid metastases from the breast cancer was positive for ER, PR, and GATA3, but negative for HER2, TTF-1, PAX8, and calcitonin. In additon, GATA-3 (+), ER (+), PAX-8 (-), and TTF-1(-) in thyroid metastases cells were reported by Magers et al. (2).

For the treatment and prognosis of thyroid metastases from breast cancer, Surgery for thyroid metastases is debatable. Some authors stated that thyroidectomy seem to be effective for alleviating dysphagia and dysphonia resulted by large metastatic thyroid lesions (5, 20, 21). Thyroidectomy was recommended for isolated thyroid metastases from breast carcinomas (9). Other cases suggested that thyroidectomy has no efficacy in patients with thyroid metastases from breast cancer combined with multiple distant metastases and chemotherapies should be performed (1, 4). With the limitation of the small number of patient data, there is no definitive evidence to support surgery or chemotherapy or radiotherapy for thyroid metastases from breast cancer (4). In addition, several studies have suggested that the prognosis of thyroid metastases is poor (9). Zhou et al. (1) reported that two of eight patients with thyroid metastases from breast cancer died at less than 22 months post-thyroid metastases diagnosis. Kim et al. (3) reported one of five patients died at 26 months after diagnosis of thyroid metastases from breast cancer. Lacka et al. (6) reported that one patient with thyroid metastases from breast cancer had metastases in liver, and lung at 5 months after thyroidectomy, and died at postoperative 36 months. Metastases in bone at 32 months after hemithyroidectomy were observed in a patient with thyroid metastases from breast cancer who died at postoperative 45 months (4). The characteristics of thyroid metastases from breast cancer are presented in **Table 1**.

**Table 1:** Reports of thyroid metastases from breast carcinoma.

Study	Year	No. of patients	Age(years)	Sex	Histology	Interval between Breast cancer and thyroid metastasis (months)	Others metastasis	Treatment	Survival time after diagnosis (months)
Kim et al. (3)	2005	5	34–55	F	Ductal carcinoma	18–85	Lung, neck LN	Chemotherapy	4–26
Owens et al. (10)	2005	1	64	F	NA	5	Liver, shoulder	NA	NA
Zhou et al. (1)	2019	8	43–69	F	Adenocarcinoma, Ductal carcinoma, Signet ring cell carcinoma,	6–82	Chest wall, Lung, Cervical and Mediastinal LN	Chemotherapy, total thyroidectomy, Right lobectomy	4–45
Egana et al. (11)	2012	1	83	F	Infiltrating lobular carcinoma	3	Live and bone	Left lobectomy	1
Lacka et al. (6)	2012	1	54	F	Ductal-lobular carcinoma	14	Live, lung	Total thyroidectomy	3
Bourcier et al. (19)	2018	1	54	F	Lobular carcinoma	NA	Cervical LN	Total thyroidectomy and cervical lymph node dissection, Endocrine therapy	NA
Pensabene et al. (4)	2018	1	64	F	Infiltrating lobular carcinoma	6	Bone	Left lobectomy	45
Ghias et al. (5)	2019	1	67	F	Ductal carcinoma	NA	Right cerebellopontine angle	Right lobectomy	NA
Present study	2021	1	54	F	Ductal carcinoma	22	Cervical LN, chest wall	Chemotherapy	NA

F, female; LN, lymph node; NA, Not available.

## Conclusion

We reported the rare case of thyroid metastases from breast carcinoma, which highlights that thyroid metastases should be considered in a patient with thyroid lesions in combination with a history of breast carcinoma. FNAB and IHC may contribute to distinguish thyroid metastasis from primary thyroid cancer.

## Data Availability Statement

The original contributions presented in the study are included in the article/supplementary material. Further inquiries can be directed to the corresponding author.

## Ethics Statement

This study was approved by the ethics committee of the West China Hospital, Sichuan University, and written informed consent was provided by the patient for publication of this case publication.

## Author Contributions

YW and SZ contributed equally to this work and are co-first authors. ZL, JZ, and TW designed the research. YW, SZ, BY, and PZ developed the literature search. YW and SZ drafted the article. All authors contributed to the article and approved the submitted version.

## Funding

This work was supported by the Sichuan Science and Technology Program (No. 2019YJ0038).

## Conflict of Interest

The authors declare that the research was conducted in the absence of any commercial or financial relationships that could be construed as a potential conflict of interest.

## Abbreviations

FNAB, fine needle aspiration biopsy; US, ultrasound; IHC, immunohistochemistry; GATA3, GATA-binding protein 3; HER2, human epidermal growth factor receptor 2; ER, estrogen receptor; PR, progesterone receptor; TTF1, thyroid transcription factor; TG, Thyroglobulin; GCDFP15, gross cystic disease fluid protein 15; CT, computed tomography; PAX8, paired box 8; HE, hematoxylin and eosin.
